# The Challenges of Recombinant Endostatin in Clinical Application: Focus on the Different Expression Systems and Molecular Bioengineering

**DOI:** 10.15171/apb.2017.004

**Published:** 2017-04-13

**Authors:** Abbas Mohajeri, Sarvin Sanaei, Farhad Kiafar, Amir Fattahi, Majid Khalili, Nosratollah Zarghami

**Affiliations:** ^1^Department of Biotechnology, Zahravi Pharmaceutical Company, Tabriz, Iran.; ^2^Tuberculosis and Lung Disease Research Center, Tabriz University of Medical Sciences, Tabriz, Iran.; ^3^Department of Clinical Biochemistry and Laboratory Medicine, Faculty of Medicine, Tabriz University of Medical Sciences, Tabriz, Iran.; ^4^Department of Basic Science, Maragheh University of Medical Sciences, Maragheh, Iran.; ^5^Department of Medical Biotechnology, Faculty of Advanced Medical Sciences, Tabriz University of Medical Sciences,Tabriz, Iran.

**Keywords:** Endostatin, Angiogenesis, Expression system, Bioengineering, Molecular targeted therapy

## Abstract

Angiogenesis plays an essential role in rapid growing and metastasis of the tumors. Inhibition of angiogenesis is a putative strategy for cancer therapy. Endostatin (Es) is an attractive anti-angiogenesis protein with some clinical application challenges including; short half-life, instability in serum and requirement to high dosage. Therefore, production of recombinant endostatin (rEs) is necessary in large scale. The production of rEs is difficult because of its structural properties and is high-cost. Therefore, this review focused on the different expression systems that involved in rEs production including; mammalian, baculovirus, yeast, and Escherichia coli (E. coli) expression systems. The evaluating of the results of different expression systems declared that none of the mentioned systems can be considered to be generally superior to the other. Meanwhile with considering the advantages and disadvantage of E. coli expression system compared with other systems beside the molecular properties of Es, E. coli expression system can be a preferred expression system for expressing of the Es in large scale. Also, the molecular bioengineering and sustained release formulations that lead to improving of its stability and bioactivity will be discussed. Point mutation (P125A) of Es, addition of RGD moiety or an additional zinc biding site to N-terminal of Es , fusing of Es to anti-HER2 IgG or heavy-chain of IgG, and finally loading of the endostar by PLGA and PEG- PLGA nanoparticles and gold nano-shell particles are the effective bioengineering methods to overcome to clinical changes of endostatin.

## Introduction

Angiogenesis is multi step formation of new blood vessel from the pre-existing ones and circulating endothelial precursors.^1^ It is based on controlled dynamic mechanism that can happen physiologically in those tissues that undergo active remodeling in reaction to hypoxia and stress. Angiogenesis plays an essential role in physiological processes such as embryogenesis, tissue growing and regeneration.^1,2^ Also, it has a critical role in rapid growing and metastases of the tumors through supplying oxygen and nutrients for cancerous cells.^3^ Angiogenesis leads to some diseases including; rheumatoid arthritis, cancer and heart disease.^4^ Many stimulants of angiogenesis have been reported including; matrix-degrading enzymes, bioactive lipids, number of small molecules, cytokines and growth factors.^5^

Therefore, considering the numerous studies on angiogenesis in recent years, inhibition of angiogenesis is a suitable strategy for molecular target therapy of cancer.^6^ Current trend is focused on the application of anti-angiogenesis agents (endogenous or synthetic drugs) for preventing the progression of malignant tumors. The first anti-angiogenesis therapy was discovered about 40 years ago.^7^ In the recent years, the several molecules discovered to display antiangiogenic activity has exponentially increased. The continuing discovery of antiangiogenic molecules has occurred in four phases. The first phase of research yielded small antiangiogenic molecules; for example, carboxy-amino-triazole, TNP-470 and protamine. The second phase was considered by finding that circulating polypeptide growth factors/cytokines including; interferon-α, thrombospondin, and platelet factor 4 that could also display antiangiogenic function. The third phase of discovery yielded fragments of proteins (themselves inactive as angiogenic inhibitors) such as Endostatin, Angiostatin, Arresten, Canstatin, Heparin-binding fragment, Kringle 5, Kringle 1–5, PEX, PF-4, Prolactin, Restin, TSP-1, Tumstatin, Vasostatin and Vastatin.^8^ Finally, within the last five years, about ten novel endogenous proteins with antiangiogenic activity were discovered for example FKBPL, CHIP, ISM1, MMRN2, ARHGAP18, ZNF24, GPR56 TAp73, SOCS3 and JWA.^4^

Cancer therapy usually has targeted tumor cells, whereas treatment with antiangiogenic agent focuses on the endothelial cells of tumor blood vessels.^8^ The angiogenic suppressors act based on the angiogenic network including; (1) Increasing of the secretion of anti-angiogenic factors, (2) Suppression of the stimulation of other endothelial cells and macrophages by the tumor cells, (3) Suppression of vital proteases that are involved in new vessel formation by endothelial cells (4) Prevention or decreasing of the emission of angiogenic agents by tumor cells, (5) Affecting the VEGF activities, (6) Suppression of endothelial cell (EC) survival ,(7) Inducing the EC apoptosis and (8) Making the resistance endothelial cells to angiogenic agents.^3^

Thalidomide was approved for multiple myeloma in Australia for the first time; later, Avastin was approved as an anti-angiogenesis drug for treatment of colorectal cancer in U.S.A.^2^ These achievements were new hopes for targeted therapy of cancer. Unfortunately, there were some problems with Avastin and other Anti-VEGF monoclonal antibodies. For example mutant tumor cells may produce many angiogenic factors and affect long term use of the drugs.^3^ Therefore, it was critical to develop low-toxic and broad spectrum agents. As a next generation of anti-angiogenesis agent, rEs holds promise for safe targeting of angiogenesis in different cancers. Endostar (N-terminal modified rhEs) was permitted by the State Food and Drug Administration in China as a specific drug in non-small cell lung cancer therapy.^9^ Non-small cell lung cancers, which are 85–90% of lung cancers, are the most prevalent malignancy after non-melanocytic skin cancer and deaths from lung cancer is more than other types of cancer.^10^ Recombinant Es suppresses different types of tumors with low toxicity and drug resistance in long term application. Also, endostatin down-regulates the abnormal angiogenesis by modifying 12% of the human genome. In addition, it does not have toxicity and does not lead to the development of drug resistance in clinical studies, even in continuous treatments.^2^ This review focused on the current knowledge about Es, different expression systems involved in its production and variety of its molecular bioengineering.

### Source of Endostatin

Endostatin is a protein with a relative molecular weight (Mr) of 20 kDa that was first isolated in 1996 from murine hemagioendothelioma (EOMA) cell culture medium.^11^ Endostatin is cleaved from the C-terminal of collagen XVIII during proteolysis mechanisms by various proteinases, including elastase, procathepsin L and matrix metalloproteinases (MMPs). Collagen XVIII is a protein located in most basement membranes (BMs) in the body such as vascular basement membrane.^12^ Proteolysis of collagen XVIII leads to production of endostatin monomers and NC1 trimers in vivo which these forms can be identified from serum and tissues.^13^ Collagen XVIII at NC1-domain consists of an N-terminus association domain (about 60 amino acids) that is followed by a triple helical domain and 180-residue of endostatin domain. A flexible hinge area including several protease-sensitive sections joins the N- and C terminus, and cleavage at this site will result in releasing of endostatin from type XVIII collagen.^14^ MMPs and cathepsin L are involved in the generation of larger fragment (30 kDa) and endostatin fragment (20kDa), respectively.^13^ Endostatin is degraded by the proteinases except MMPs and its generation and stability is regulated by peri-cellular protease.^15^

### Structure of Endostatin

**Figure 1:**
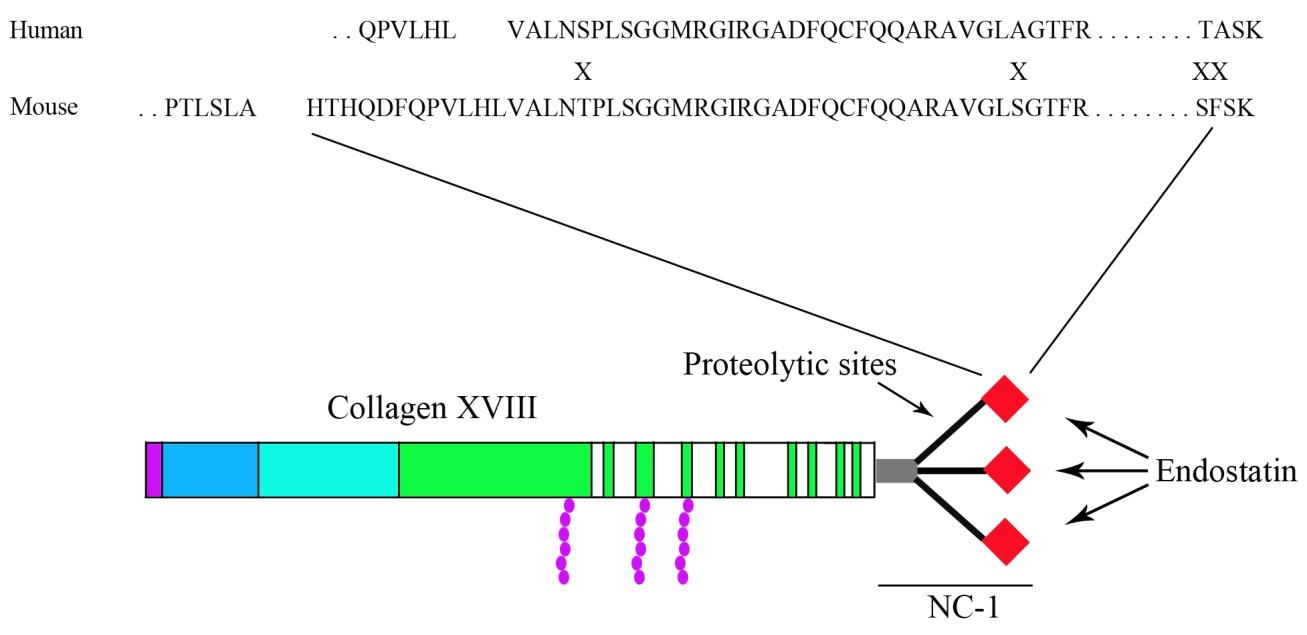
The 3-structure of human endostatin. The β-strands are yelow, α helix is red and connecting loops are blue. The cyctines that are involved in disulfide bound formation are green and the zinc is orange. This Schematic structure was obtained from the NCBI site (MMDB 3D structure).

**Figure 2:**
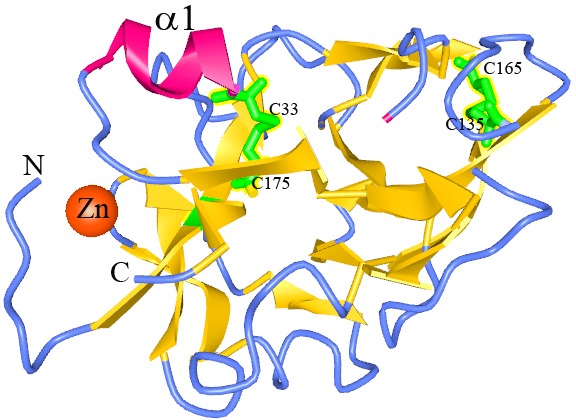
Schematic structure of type XVIII collagen, Non-collagenous (NC) domain, trimerization domain and protease sensitive sites. The sequences from the non-triple-helical C-terminal regions of mouse endostatin and human circulating endostatin indicate high hemology between mouse and human endostatin and the mismatches between these sequences are showed by X. The sequences alignment was obtained from the L. Staëndker et al. study (21).

Endostatin is a C-terminal 184 amino acid (132–315 AA) part of collagen XVIII, including 40% highly hydrophobic residues that lead to formation of a large hydrophobic core by many surface loops. Endostatin also has 29 basic amino acids, 16 acidic amino acids 15 arginine residues and four cysteines forming two disulfide bridges.^16,17^ High-resolution X-ray studies on this protein have revealed that it has a compact globular structure containing b-sheets and loops, two α helices, two disulfide bridges, heparin binding site and a Zn (II)-binding site at the N-terminal.^14,18,19^ The bond Cys_33_-Cys_173_ joins the focal β-sheet to the longer α-helix (Figure 1).^17^ His_1_, His_3_, His_11_, and Asp_76_ are the ligands of Zn (II) in human endostatin (hEs) that are conserved in dog and murine endostatin. His_1_, His_3_, and Asp_76_ are essential for Zinc binding in Es. The site mutation in His_11_ or Asp_76_ also notably decreases the antitumor activity of full length Es.^20^ Human endostatin shows about 86% of identity and >90% similarity with mouse endostatin (mEs) sequences (Figure 2)^21^representing a high structural (and probably functional) relationship. The alignment of mEs (from tumors) and hEs from circulation revealed that hEs has 12 amino acids less than other one. The hEs lacks a single lysine amino acid in C-terminal, demonstrating possible processing by carboxypeptidase(s). The difference between hEs and mEs might be explained by its production from different origins.^17^

### The structure- function relationship 

The zinc-binding site of hEs is close to the N-terminal that is formed by three histidines (H1, H_3_ and H_11_ at the N-terminal) and an aspartic acid at residue 76.^22^ The zinc binds to Es at a 1:1 molar ratio.^23^ *Bohem* et al. (1998) supposed that the ability of Es to bind zinc is critical for its antiangiogenic function via single point mutation in His1 and Asp3.^24^ But, *Cho* et al. and *Chillem* et al. reported that N-terminus deleted mutant of hEs act the same as wild-type hEs.^25^ In following, it was reported that zinc binding is essential for antimigration and antitumor activities of hEs but not its antipermeability property.^26^ Finally, it was established that the binding of zinc to Es considerably increase stability of the Es.^18^ The addition of an extra zinc binding domain at N-terminal of hEs lead to increase thermal and proteolytic resistance of Es.^20^

The heparin-binding sites are formed by 11 noncontiguous arginines that cluster together over the surface of the Es at two sites, firstly (around R_158_ and R_270_) and secondary binding site (R_193_ and/or R_194_). But, an effective binding of Es to heparin needs a synchronous binding of a single heparin molecule to both sites.^19^ Endostatin needs to both the minor and the major heparin-binding site for inhibiting FGF-2, whereas for inhibiting of VEGF- A needs to the minor heparin-binding site in its anti-angiogenesis activity.^27^

Disulfide bonds are formed by Cys_33_-Cys_173_ and Cys_135_-Cys_165_ in mEs and hEs.^28^ The presence of two disulfide bonds, which is the result of post-translational modification, results in a highly folded structure that makes be acid-resistant. Disulfide bonds are critical for the structural compactness, stability and biological function of the Es. Eliminating of disulfide bonds by site mutation resulted in fibrillar aggregates form of Es even at near neutral pH.^29^

#### The molecular mechanism of action

Despite extensive studies of physiologic effects of Es on angiogenesis and tumor growth, its molecular mechanism is a matter of debate yet. The anti angiogenic activity of Es are not the result of single molecular action, but very convoluted. Several studies have been done to describe the anti angiogenic activity of Es and recognize the nature of the Es binding partners. Es activity leads to inducing apoptosis of endothelial cell and stopping the cell cycle, and suppresses endothelial cell proliferation and migration via a complex system of signaling. It was also reported that Es powerfully suppresses the neovascularization. Endostatin’s antiangiogenic activity can be predicted by several mechanisms including: inducing the endothelial cell apoptosis; inhibiting the endothelial cell proliferation and migration; inhibiting the actions of angiogenic inducers; affecting the activity of protease and affecting the angiogenic signaling pathways.

#### Induction of the apoptosis in endothelial cell 

Endostatin activity leads to apoptosis of endothelial cells, but has no effect on various normal, transformed or neoplastic cells. Therefore, it seems that endostatin activity specifically affects the endothelial cell. It was established that endothelial cell apoptosis is mediated by following mechanisms of Es action:

**(I).** Anti-apoptotic proteins including Bcl-Xl, Bcl-2 and Bad inhibit apoptosis of cell in reaction to several stimuli. Conversely, Bak and Bax as pro-apoptotic proteins accelerate cell apoptosis.^30^ Researchers have established that the action of Es leads to down regulation of Bcl-2, Bcl-Xl and Bad proteins expression, and their phosphorylation status. Also, these effects of Es are not fond in several non-endothelial cells.^31-33^ Activation of Caspase-3 as an intracellular protease during apoptosis results in starting of cellular breakdown; degrading specific structural regulatory and DNA repair proteins. It was reported that addition of Es up regulates the caspase- apoptotic pathways that results in degradation of DNA in the nucleus.^32^ In addition another study showed that Es activates pro-apoptotic pathways via induction of caspase-9 activation by decreasing the level of the anti-apoptotic proteins including; Bcl-2, Bcl-Xl and Bad.^31,33^

**(II).** The expression of numerous gens which are important to angiogenesis are affected by Wnt/b-catenin signaling pathway, specifically cyclin D1. Endostatin decreases the expression of cyclin D1and in turn inhibits cell proliferation and cellular migration. In addition, Es down regulates b-catenin and subsequently suppresses Wnt/b-catenin signaling pathway.^34-36^ Moreover, Es has inhibitory effect on vascular endothelial growth factor (VEGF) expression which may results in suppressing of Wnt/bcatenin signaling pathway.^35^

**(III).** Endostatin induces tyrosine kinase activity that leads to formation of multi-protein signaling complexes in endothelial cells. Shb adaptor protein is one member of these complexes, which is involved in apoptosis. Apoptosis is mediated via expression of Shb with a functional Src homology2 (SH2) domain and the heparin-binding ability of Es. Es bound to the endothelial cell surface via its heparin-binding domain and so induces tyrosine kinase activity which may lead to apoptosis.^37^

**(IV).** Endostatin interacts with tropomyosin-containing microfilaments that results in inhibition of cell motility, an induction of apoptosis and finally inhibition of angiogenesis.^38^

#### Inhibition of the endothelial cell proliferation and migration 

Nucleolin is a cell surface receptor of Es, which has vital role in the anti-angiogenesis and antitumor activities of Es. In angiogenic conditions, extracellular matrix proteins and VEGF transfer the Nucleolin from nucleus to endothelial cell surface. Endostatin specifically bound to the nucleolin and nucleolin acts as a shuttle protein in transporting of Es into the endothelial nucleus. Endostatin inhibits the phosphorylation of nucleolin in the nucleus, which is essential for cell proliferation.^39,40^

Various integrins including α_5_β_1_ and α_5_β_5_ play critical roles in endostatin function. Endostatin bind to α_5_β_1_ on the surface of proliferating endothelial cells and decreases cell migration. This action, in particular, is caused by inhibition of signaling pathways mediated by small kinases Ras and Raf.^41^ In addition, Es binds simultaneously with α_5_β_1_ and caveolin-1 that this, in turn, activates non-receptor tyrosine kinases (Src tyrosine kinase). Src tyrosine kinase is involved in the regulation of cell proliferation and differentiation and also of cell mobility. This action of endostatin leads to suppressing of endothelial cell mobility and the interactions of cell matrix.^42^ Additionally, Es quickly down-regulates various genes in exponentially growing endothelial cells. Endostatin effectively represses endothelial cell migration partly through inhibition of MAPKs and down-regulation of c-myc expression.^43^

#### The Prevention of angiogenesis induction 

FGF-2 and VEGF are two of the best-characterized proteins in stimulating angiogenesis. In order to efficiently inducing of angiogenic signaling, presences of proteoglycans as cofactors are critical for FGF-2 and VEGF function. Endostatin inhibits FGF-and VEGF-mediated migration via binding to glypican or their high-affinity receptors.^17^ The direct interaction of Es with the VEGF-R2/KDR/Flk-1 receptor leads to blocking of angiogenic function of VEGF. Also, Es decreased the activation of p38MAPK, ERK and p125FAK.^42^

#### The effect on the protease activity 

These functions of Es are most likely related to its binding to cell surface receptors and triggering series of intracellular signaling cascades such as matrix metalloproteinase 2 (MMP-2). MMP-2 mediates selective proteolytic degradation of the extracellular matrix that is essential for migration and invasion of endothelial cells at the beginning of angiogenesis. Endostatin forms a stable complex with MMP-2 via binding to its catalytic domain and inhibits its action by covering catalytic domain.^44^

#### The effect on the angiogenic signaling pathways

Endostatin down regulates various signaling pathways, which they associate with pro-angiogenic activity. Down regulation of cyclin-D1, c-myc, inhibition of MMPs and blockade of VEGF/VEGF receptor signaling are examples of how Es affects the signaling pathways. The endostatin signaling pathways consist of (1) HIF1-α, (2) Id1 and Id3, (3) NF-kB, (4) Ephrins and TNF, (5) Coagulation cascades and Adhesion Molecules, (6) AP-1, (7) Ets-1 and (8) STATs. These signal pathways causes cell cycle arrest, apoptotic induction and decreasing motility of endothelial cells. Endostatin suppresses the inducers of angiogenesis via up-regulating their antagonists. For instance, endostatin down regulates HIF-1α by up-regulating HIF-1AN (antagonist). The reduction of critical factors including Ets-1, NF-κB, STATs and HIF-1α which are upstream regulators of anti-apoptotic genes (c-myc and Bcl-2) could define the pro-apoptosis effect of Es. In addition, NF-kB, AP-1 and STATs supports proliferation by regulating cyclins like cyclin D1.^45^

## Clinical application challenges

The importance of Es leads to further studies on this protein than on any other endogenous angiogenesis inhibitor.^2^ The majority of therapeutic researches have used purified form of endostatin. There are, however, many challenges in clinical application of recombinant endostatin (rEs). First, tumor treatment needs to large quantities of biologically active Es and this, in turn, increases the importance of production of bioactive rEs in large quantities which, is difficult and has high cost.^10,46-48^ Second, protein production system may disable in producing soluble protein and correct folding of protein which affects its bioactivity.^49^ Third, the endostatin-purification process may denature its structure and the resultant yields may be low.^50^ Forth, Es has short half-life and is instable which this increases the importance of improving its in vivo stability.^51^ Finally, the necessity to deliver Es on a chronic basis may result in practical difficulties in clinical conditions.^52^

In order to produce soluble and functional endostatin in large quantities, selection of suitable expression system is considered as an important solution.^53,54^ Also, to overcoming its stability and time-acting limitations, molecular bioengineering of protein and improving tumor- specific delivery systems are the preferable approaches.^52,55^ Moreover, one probable approach to overcoming some of these problems may be the utilization of a gene therapy strategy.^52^ In following, this review will focus on different expression systems of Es protein and molecular bioengineering of endostatin that were used by several studies during recent decades.

### Systems for producing recombinant Endostatin

#### Mammalian expression system

Mammalian expression systems are commonly used for producing proteins which require to complex post-translational modifications (PTDs). The main cell types are used in this system include Chinese hamster ovary (CHO) cells, various mouse myelomas such as NS0 murine myeloma cells, baby hamster kidney (BHK) cells, insect SF-9 cell line, green monkey kidney cells and human cell lines such as human embryonic kidney (HEK) cells.^56^ The mEs and tagged-fusion-mEs (his_6_-mEs) were produced by CHO cells via inserting cDNA of endostatin in upstream of the amplifiable marker gene, dihydrofolate reductase (DHFR). The amount of secreted mEs and his_6_-mEs were 78 and 114 µg/mL, respectively that seems histidine tag increased the expression of Es. Both rmEs and his_6_-mEs inhibited endothelial cell proliferation, in a dose dependent manner.^57^ In spite of these advantages, this system has some disadvantages including: high cost, laborious production, time-consuming, poor secretion and also potential for contamination of the product by viruses.^58^

#### Baculovirus expression system (Insect cell expression system)

Insect cell expression systems have the best machinery for the folding of mammalian proteins. So they are quite appropriate for producing soluble proteins of mammalian origin.^59^ O’Reilly et al. produced rmEs using baculovirus expression system. They infected *Spodoptera Frugiperda 21* cells by baculovirus.^11^ In following, another insect cell system was applied to improve the quantities of produced soluble rEs‏. The rmEs was expressed in *Drosophila melanogaster S2* cells. The purification yield from stably transformed S2 cells was approximately 0.5 mg/L from the medium culture. In a T-flask, the stably transformed S2 cells produced 3.4 mg /L rEs. The amount of Es in this method was higher than the quantity (1-2 mg/L) that was reported from baculovirus-infected *Spodoptera frugiperda 21* cells. In a different work, Park et al. used high aspect rotating-wall vessel which was designed by NASA and increased the production up to 13 mg/L.^11,60^ The second investigation was directed by Park et al. to improve the previous study and the effect of cadmium was evaluated in inducing metallothionein promoter in the Drosophila cell-expression system. Also, they added Drosophila BiP protein signal sequence to their construct for increasing of extracellular secretion. Their results indicated that the secreted rEs was approximately 89% of the total rEs. The optimal production of rEs was about 2.5 mg/L. Recombinant endostatin production was increased up to 17 % (3mg/L) by using Sodium butyrate supplementation expression strategy.^61^ However beside these advantages, insect cell systems have some limitations including: poor expression, unusual glycosylation and requirement for evaluation of particular patterns of post-translational modification.^56^

#### Yeast expression system

Yeasts, the single-celled eukaryotic fungal organisms, are commonly applied to production of recombinant proteins that are not be able to be expressed well in *E.coli* because of difficulties such as mis-folding or lack of glycosylation. The major advantages of yeast expression systems include; high yield, stable production, cost effectiveness, mammalian-like PTMs, proper folding of S–S rich proteins and high density growth. Also, yeasts have some considerable benefits compared to insect or mammalian cells. They are easier to handle, less expensive and are easily adaptable to fermentation processes.^56^ The three most utilized yeast strains are *Saccharomyces cerevisiae* (*S. cerevisiae*), *Pichia pastoris* (*P. pastoris*) and *Hansenula polymorpha* (*H. polymorpha*). However, glycosylation by *S. cerevisiae* is often unacceptable for mammalian proteins because the O-linked oligosaccharides contain only mannose; furthermore, it may cause immunological reactions against the produced recombinant proteins in the body. For overcoming these problems, the methylotrophic yeasts can be used instead of *S. cerevisiae* for production of recombinant proteins. Methylotrophic yeasts are cost effective hosts and they can produces high levels of recombinant proteins with considerable stability.^62^ Endostatin has two disulfide bonds in its structure that is essential to its folding and function. One of the best advantages of *P. pastoris* over *E. coli* is its ability to produce disulfide bonds of proteins. This means that in this situation where disulfides are important, *E. coli* might produce a misfolded protein, which is usually inactive or insoluble. Also, Pichia usually gives much better yields, compared to other expression systems such as Chinese Hamster Ovary (CHO) cells or S2-cells from Drosophila melanogaster.^63^ Additionally, production of recombinant proteins such as endostatin in *P. pastoris* is more economical. One of the most notable properties for expression systems is having strong and strictly adjustable promoter that methylotrophic yeasts have these advantages.^62^ Because of low yield of endostatin expression that is reported in mammalian expression system, researchers focused on yeast expression system.^15,22^ For the first time, Dhanabal et al. used yeast expression system for production of mEs and hEs respectively and they used *P. pastoris*. They firstly achieved 15-20 mg/L mEs and then 10 mg/L for hEs from yeast culture. They reported that the expressed Es in this expression system causes G1 arrest of endothelial cells, induction of apoptosis in HUVE and HMVE-L cells, and these effects do not occur in non-endothelial cells.^64^ In *P. pastoris*, KEX1p gene cods for an enzyme that able to cleave arginine and lysine residues from the C-terminus of peptides and proteins. Boehm et al. disrupted the *P. pastoris* KEX1 gene to overcome this deficiency. Results showed that their method could produce full length of murine endostatin and human endostatin in *P. pastoris*.^65^ These are three types of recombinant Pichia including: Mut^+^ where the two methanol oxidases genes AOX1 and AOX2 are intact; Mut^S^, where only AOX2, which is responsible for 15% of the protein biosynthesis, is intact; and Mut^−^ where both AOX1 and AOX2 genes are disrupted. The Mut^+^ strain is the most commonly used recombinant strain and is the most responsive one to methanol concentration.^66,67^ In 2002, Li et al. improved the rhEs production in fed-batch cultures of *P. pastoris* using the methanol feeding rate. *P. pastoris* Mut+ phenotype clone was used while both AOX1 and AOX2 genes were intact. They applied constant methanol feeding strategies during the induction phase, and examined the effects of different constant methanol feeding rates on post-induction cell growth and rhEs protein production. Their findings indicated that the methanol feeding rate could be used to guide parameters for large-scale production of recombinant proteins in the *P. pastoris* system.^68^ In 2002, Trinh et al. evaluated effect of methanol feeding strategies on production of rmEs and yield rmEs from *P. pastoris*. They used three methanol addition strategies including: two strategies for the yeast metabolism that the first was responding to the methanol consumption and the second was responding to the consumption of oxygen; the third one was based on a predetermined exponential feeding rate. Total production level of Es in three methods was similar (400 mg from 3L initial volume), but in the third method, amount of the added methanol and produced biomass were lower. Therefore the third method was more efficient and more suitable for downstream processing.^66^ Translational inefficiency is one of the reasons that could limit Es production in yeast expression system such as *P. pastoris*. Synonymous codon usage bias differences are the most probable causes of the observed translational obstacles.^69,70^ In 2007, Su et al. applied artificially synthesized construct according to mature peptide sequence in human collagen XVIII for overcoming this problem, they optimized 20 codons of Es gene construct for expressing in *P. pastoris*. This construct was successfully expressed in *P. pastoris* and SMD1168 strain was selected as a strain that had high-yield expression of endostatin high-density fermentation. The amount of produced recombinant protein in culture media was 80 mg/L in shake flask cultivation and 435 mg/L in high-density bioreactor fermentation.^71^ Paek et al. expressed two soluble fusion proteins, human Angiostatin (hAs) and hEs in *P. pastoris* and evaluated whether their orientation affected their antiangiogenic properties, through the use of the VEGF promoter assay. Their results revealed that the orientation of the fusion genes in hAs and hEs might be an important factor in the development of therapeutic proteins.^72^*H. polymorpha* is the other methylotrophic yeast that was used by Wu et al. for producing rhEs. Despite the substantial similarities of methylotrophic yeasts, *H. polymorpha* system shows some unique features that distinguish it from the *P. pastoris* system. For example, *H. polymorpha* is more heat tolerable (30–43 °C) than *P. pastoris* and possesses the methanol oxidase promoter (P_MOX_) instead of P _AOX1_ that leads to *H. polymorpha* to be derepressed at low glycerol concentration. These properties make *H. polymorpha* an appropriate expression system for the industrial-scale production of recombinant proteins.^73^ Their research evaluated the possibility of *H. polymorpha* as an alternative expression system for Es production. They achieved 65mg/L of rhEs in shake flask.^74^ There are however, some deficiencies of using *P. pastoris* as a host for heterologous expression. A number of proteins require chaperones for correct folding. *P. pastoris* cannot produce such proteins. Gerngross et al. designed a strain of *P. pastoris* that produced that its glycosylation was similar to human glycosylation form.^75,76^ They exchanged the enzymes which were responsible for the yeast type of glycosylation. Therefore‏, this different glycosylation pattern permitted to production of a fully functional protein. This strain of engineered *P. pastoris* has been applied in the production of other recombinant proteins.

#### Escherichia coli expression system

*Escherichia coli* is a microorganism that is one of the earliest hosts and extremely useful for production of heterologous proteins with commercial interest at large scale for structural and functional studies.^77,78^*E. coli* genetics are far better understood than those of other microorganisms. Advanced genetic tools convert this bacterium to a valuable host for expression of complex eukaryotic proteins. Moreover, ability of rapid growth, high expression level, avoidance of incorporation of amino acid analogs, formation of intracellular disulfide bonds, alteration of metabolic carbon flow and cheapness are the other advantages of this system.^56,79^ O’Reilly et al. used *E. coli* expression system beside the insect expression system, for the first time. They obtained insoluble murine recombinant endostatin and compared this protein with native form derived from collagen XVIII and recombinant protein that was expressed in insect expression system. They declared that the inhibition of endothelial cell proliferation was similar in all of the three derived forms. More than 99% of the protein derived from *E. coli* was insoluble and lost during centrifugation.^11^ Generally, protein concentration and the pH of refolding buffer are very essential factors in the refolding process of recombinant proteins.^80^ In this point, for overcoming the production of insoluble rEs, You et al. designed an applied new method for refolding and purification of murine endostatin in *E. coli* system for the first time in 1999. For this purpose, they refolded the protein in the presence of chaotrophic agent (urea and guanidine-Hcl) and redox-coupling reagents such as glutathione. This protein was comparable to the one obtained from the use of Yeast-expression system in physiochemical properties and also in anti-angiogenic activities.^81^ In another study, production of soluble recombinant murine endostatin was the main aim of study. A purification method was developed by Huang et al. in order to production of an effective and soluble rEs. Their study was resulted in production of 150 mg/liter-culture and 99% purity of recombinant murine endostatin.^82^ Another strategy to produce soluble recombinant protein is using secretion system of *E. coli*. This system provides soluble protein with high biological activity and easier purification.^83^ Xu et al. utilized secretion system. They placed alkaline phosphatase gene (phoA) promoter in downstream of a murine endostatin gene. For folding and secretion of the recombinant murine endostatin, they fused the interest gene with alkaline phosphatase signal peptide sequence. They selected this signal peptide because it can produce biologically active heterologous proteins in large scale and can be correctly cleaved. Their study showed that this system could produce about 40 mg/L endostatin in the fermentation broth.^84^ Following the previous study, Xu et al. produced soluble and bioactive rhEs in *E. coli* by employing Trigger factor (TF) and the other two groups of molecular chaperones. They evaluated effects of different temperatures or different induction duration beside co-expression of GroEL/Es with DnaK-DnaJ-GrpE and Trigger factor on the production of soluble and active rhEs. Their result showed that low temperature or molecular chaperones alone could increase the production of bioactive rhEs. Applying low temperature cultivation (16°C) with co-expression of DnaK-DnaJ-GrpE and GroEL/Es was more impressive for prevention of the rhEs aggregation. The yield of soluble rhEs was about 36mg/L‏, and at least 16mg/L of recombinant human endostatin was purified.^85^ In 2008, Chura-Chambi et al. optimized new methods for solubilizing and refolding of insoluble Es aggregated as inclusion bodies (IBs). They applied High hydrostatic pressure (200 MPa or 2 kbar) in addition to guanidine hydrochloride in the presence of reducing and oxidizing agents. They obtained about 90 mg/L recombinant murine endostatin.^86^ One of the challenging problems for clinical application of rEs produced from bacteria is multimer formation of Es. In this point, Wei et al. studied the multimer and monomer structures efficacy on bioactivity of rEs produced in *E. coli*. They suggested that multimer endostatin had a comparable or higher bioactivity and its bioactivity not be affected by multimerization.^87^ DuC et al. studied the effects of glutathione S-transferase (GST) and NusA protein on solubility of rhEs in *E. coli*. They revealed that NusA protein increased the production of soluble rhEs; but GST did not affect the amount of produced rhEs.^88^ Yari et al. for solubilizing of recombinant murine endostatin fused it with thioredoxin. They showed thioredoxin could enhance the solubility of protein and simplify its purification.^79^ Chura-Chambi et al. with the aim of increasing the efficiency of Es refolding under pressure, analyzed the factors that are involved in the dissociation of IBs and the refolding of Es, these factors are as the followings: (i), the impact of the growth temperature (25°C) on the quality of aggregated endostatin. (ii), effect of high hydrostatic pressure (2.4 Kbar) with subzero temperatures (-9°C) on the dissociation of endostatin. (iii), the use of small molecule additives (such as1.5 M Gdn-Hcl) to increase the recovery yields of native Es in association with the action of the pressure. Their findings suggest that application of 2.4 Kbar and 9°Cdissociated of the IBs produced at 25 °C and also presence of 1.5 M Gdn-HCl could enhance the efficacy of dissociation.^89^

The level of rEs, in different expression systems, is summarized in Table 1.

### Molecular bioengineering 

The short half-life and in vivo instability are the limitations of endostatin in clinical application which, in turn affects its bioactivity. These limitations can be overcome via molecular modifications including single point mutation, addition of a zinc binding site, fusing of rEs to antibodies or suitable protein carriers.^90-93^ Also, for further increasing of stability, slow release formulations can be used.^94,95^

**Table 1:** The level of recombinant Endostatin in different expression systems.

**Host Cell**	**Yield (mg/l)**	**Description**	**Reference**
CHO*	78	**Es	57
CHO	114	(his)6-met-Es	57
*Drosophila melanogaster S2cells*	13	using high aspect rotating-wall vessel	18
*P. pastoris*	15-20	-	64
*P. pastoris*	10	-	64
*P. pastoris*	133	Using methanol feeding strategies	66
*P. pastoris*	435	In bioreactor	71
*H. polymorpha*	65	-	74
*E. coli*	150	By developing a purification protocol	82
*E. coli*	40	Fusing Es with phoA sp	84
*E. coli*	36	Co expressions with chaperones	85
*E. coli*	90	developing solubilizing and refolding methods	86

* *Chinese Hamster Ovary cells* ** *Endostatin*

**Figure 3:**
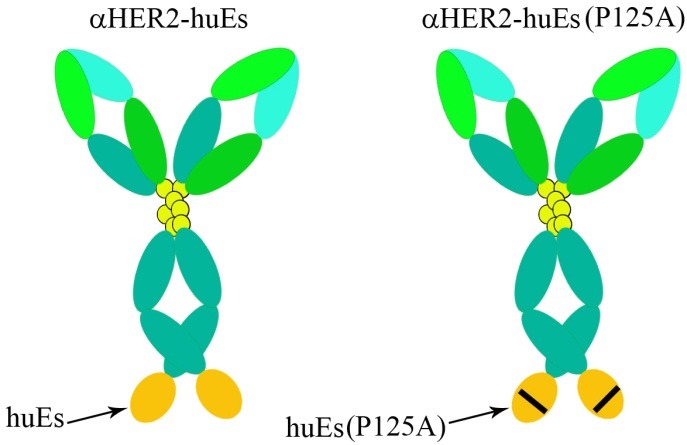
The Schematic Picture of fused hEs to anti-HER2 IgG3

**Figure 4:**
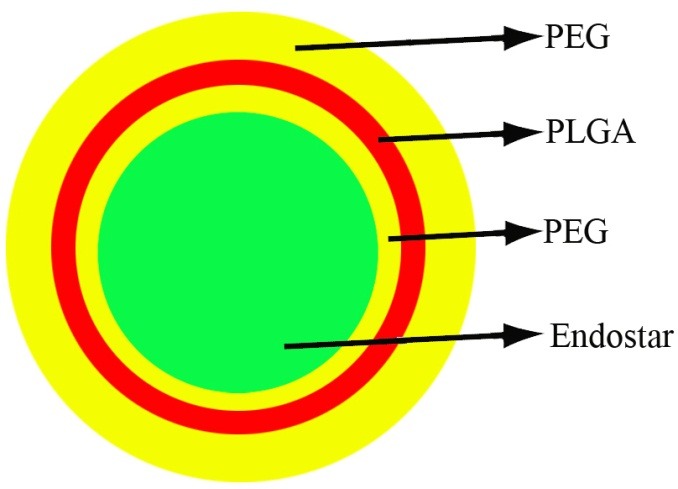
Encapsulated Endostar in PEG-PLGA nano particle.

Previous studies have shown rEs in dimers or trimers forms have more effective antitumor activity than monomeric forms. Endostatin oligomers effectively stimulate the motility of endothelial cells that can be a strategy in enhancing anti-tumor activity of rEs.^96^ A point mutation in rhEs at position 125 (P125A) is another way that enhance the anti-angiogenic activity of Es and support proper localization of rEs into tumor tissue. The synthetic peptide corresponding to the sequence 93–133 of Es displayed a pro-angiogenic activity, enhanced migration of the endothelial cell and neovascularization. The substitution of proline at 125 with an alanine may lead to a conformational modification that decreases the pro-angiogenic activity of this sequence. The internal Asn-Gly-Arg (NGR) motif of hEs is placed at position 126–128. Point mutation of the hEs at position 125 (P125A), may lead to enhancing of the tumor vascular targeting by the NGR motif and so efficient binding of the mutant endostatin to endothelial cells. Mutant endostatin displays more inhibition of both proliferation and migration of endothelial cells than native endostatin. In addition, mutant endostatin down-regulates the angiopoietin 1 and vascular endothelial growth factor more effectively than native endostatin.^91^ Jing et al., in order to improve biological activity and potency of hEs, modified mutant endostatin (P125A) via addition of an integrin-targeting moiety (RGD). Addition of RGD sequence resulted in better localization to tumor vasculature and enhanced the antiangiogenic activity of rEs. Endostatin has relatively short half-life in systemic circulation.^55^ One of the methods to enhance biological half-life of proteins in systemic circulation is conjugating it to Fc fragment of IgG. Lee et al. increased biological half-life of Es via fusing to the carboxyl terminus of human Fc heavy chain.^92^ Jing et al. showed that Es can be fused to the amino terminus of human Fc heavy chain. They designed and prepared genetically fused mutant (P125A) endostatins including an RGD sequence at the amino terminus (RGD-P125Aendostatin- Fc) or spliced between the angiogenesis inhibitor (P125A-endostatin-RGD-Fc) and Fc fragment. The results showed that RGD sequence in the fusion proteins is accessible to cell surface integrins regardless of its location either at the splice junction or at the amino terminus. The evaluation of antiangiogenic and antitumor activities showed that RGD- endostatin (P125A) has better biological activity compared to endostatin (P125A). Also Fc-fusion proteins presented more enhanced biological activity than rEs. Enhanced potency of fusion proteins could be because of the modifications that were improved by protein folding and stability.^55^ Another strategy to improve biological half-life and bioactivity of Es is fusing of Es to the anti-HER2 antibody**.** Shin et al. generated two Es fusion proteins by joining wild-type or mutant (P125A) human endostatin to the 3’ end of humanized anti-HER2 IgG3 that delivered dimeric Es (Figure 3). The result showed that fusion proteins markedly improved antiangiogenic and antitumor activity of hEs in several cancer models. Also, aHER2-huEndo (P125A) specially inhibited tumors expressing HER2 antigen. The fusion proteins had a markedly longer serum half-life than hEs alone which similar results achieved with a murine endostatin fusion protein.^93^ As previously reported mutant endostatin (P125A) displayed more antiangiogenic activity than native endostatin.^91^ In accordance with this, the aHER2-huEndo (P125A) fusion protein displayed better inhibition of tube formation in vitro than wild- type endostatin, mutant endostatin (P125A), or wild type aHER2-huEndo fusion. In addition, presentation of Es as dimers causes the endostatin efficiently bind to perlecan, glypicans and integrins and in turn, increase fusion protein activity.^96^ Adding an extra zinc binding site (MGGSHHHHH) at the N-terminus of Es was the most valuable molecular modification of rEs that lead to innovation of new specific drug, Endostar, used in treatment of non-small-cell lung cancer. Endostar was approved by the State FDA in China in 2005. This modification led to increasing thermo stability and proteolytic resistances of rhEs. Jiang et al. compared thermo stabilities and zinc-binding Endostar (holoEndostar) and zinc-free Endostar (apoEndostar).They declared that addition of the zinc binding site to rhEs increased the transition temperature towards thermally induced denaturation and improved its resistance to trypsin, chymotrypsin, CPA (carboxypeptidase A) and CPB (carboxypeptidase B).^90^ Endostar, like most of the other protein drugs has short biological half-life because of its rapid metabolism. Therefore, using a novel protein delivery carrier with well- controlled release can increase the anti- tumor activity of endostar. Also, Molecularly-targeted therapy based on nanotechnology and nonmaterial can be extremely valuable in cancer therapy. Cancer cells easily ingest the nanoparticles that cause enhancing of anti-tumor activity of drugs. So, nanoparticles can be ideal carriers in molecularly-targeted therapy.^97,98^ Chen et al. in order to find an effective carrier for the encapsulation and delivery of endostar prepared particulate carriers (nanoparticles and microspheres) of poly (DL-lactide-co-glycolide) (PLGA) and poly (ethylene glycol) (PEG)-modified PLGA (PEG-PLGA) (Figure 4). Their results were consistent with Hu et al. reports that indicated endostar-loaded PEG-PLGA nanoparticles have a better anticancer activity than conventional endostar.^94,95^ The endostar-loaded PEG-PLGA particulate carriers had the higher encapsulation rate than PLGA particulate carriers due to the presence of PEG. The hydrophilic moiety on the surface of PEG-PLGA particulate carriers cause carrier bind more easily to soluble endostar and in turn enhanced the encapsulation efficiency of PEG-PLGA particulate carriers. Also, the biodegradation rate of the microspheres and nanoparticles are dependent on the surface area of the polymer and the hydrophilic/lipophilic properties ratio. Therefore, the presence of PEG led to increasing of the release speeds of the microspheres and nanoparticles. Also, the larger surface area of the PEG-PLGA than microspheres resulted in increasing of the release speeds of the endostar-loaded PEG-PLGA nanoparticles. So, endostar-loaded PEG-PLGA nanoparticles are better than other three particulate carriers due to high encapsulation and rapidly releasing of the endostar.^94^ In another study, for targeting therapy of tumor angiogenesis, a new theranostic nanostytem was generated. Firstly, the Endostar was loaded with PLA nanoparticles (EPNPs) and then was coupled with GX1 peptide and was conjugated to the surface of EPNPs near infrared (NIR) dye IRDye 800CW, GX1-EPNPs-NIR dye IRDye 800CW (GEN) in order to monitor of the bio-distribution. GX1 peptide is a tumor vasculature endothelium-specific ligand. GEN facilitates effective release of chemotherapeutic agents to tumor site, while decrease toxicity and side effects, also facilitates to real time screen tumor targeting in vivo. The comparison of these agents showed that the tumor inhibitory effect of GEN is better than EPNPs and Endostar, because the GX1 simplified drug accumulation in the tumor sites, and also facilitates the monitoring of drug releasing in tumor regions.^99^ Luo et al. evaluated the antitumor activity of gold nano-shell particles of Endostar (G- Endostar) and indicated that enhance the inhibitory activity of Endostar.^97^ Endostar easily degrades and equally distributes to all tissues. So, selectively delivering of endostar to the lesion part may be more effective. The circum sporozoite protein (CSP) covers the malarial sporozoite and targets the liver for infection and it was established that I-plus of N terminus of CSP, which is conserved sequence with high affinity to heparin and heparan sulfate proteoglycans, specifically bind to the liver. In accordance with them, Ma et al. linked CSP I-plus sequence to the C-terminus of endostar (endostar-CSP) and reported that endostar-CSP targets the liver and inhibits the proliferation, migration, and tube formation of endostar.^100^

## Conclusion

Angiogenesis inhibition is a promising therapeutic approach in cancer therapy. In recent years, many anti-angiogenesis agents have been discovered including endogenous inhibitors.^12^ Among the endogenous inhibitors, Es is an attractive candidate as an anti- cancer protein that has been extensively studied for the cure of cancer, rheumatoid arthritis and retinopathies.^17^ The purified form of rEs protein is used in majority of therapeutic researches. Meanwhile, as a protein, its clinical application has many challenges.^50^ Selection of suitable expression system, modifying the biochemical properties of endostatin and improving delivery systems can be the most important strategies for overcoming these short comings.^52-54^

Since the discovery of endostatin, different expression systems have been applied to produce rEs. Yeasts are eukaryotic expression systems with ability of growing in high cell densities which can produce proteins larger than 50 kDa and are able to perform post translational modifications (PDMs) of proteins. The baculovirus system is a higher eukaryotic system than yeast and can perform more complex PDMs of proteins. It provides a better circumstance to gain soluble protein when it is of mammalian origin and can express proteins larger than 50 kDa. However, the baculovirus system acts slowly, is time consuming and not as simple as yeasts. The mammalian expression system is the most popular type of system for expression of recombinant glycosylated proteins. They can produce proteins larger than 50 kDa. However, selection of cell lines is usually labor and time consuming and the cell culture is stable for only a limited time. *E. coli* expression system is the cheapest, easiest and quickest expression system of proteins. However, this system cannot express very large proteins, S–S rich proteins and proteins that require posttranslational modifications. Totally, 39% of recombinant proteins are made by *E. coli*, 35% by CHO cells, 15% by yeasts, 10% by other mammalian systems and 1% by other bacteria and other systems in the industry.^101^ Producing rEs in eukaryotic expression systems with consideration of structural properties such as molecular weight, disulfide bridges, glycosylation and folding is attractive. In spite of these advantages, high cost and time consuming processes limits the application of this systems. Briefly, none of the mentioned systems can be considered to be generally superior to the other. Meanwhile, with considering the advantages and disadvantage of *E. coli* expression system compared with other systems beside the molecular properties of Es, *E. coli* expression system can be a preferred expression system for expressing of the Es in large scale. Also, based on expression system properties and interested production level, the most proper expression system has to be identified and optimized individually for the production of rhEs.

As bioactivity and stability limit the clinical administration of rhEs, it is important to increase its bioactivity and stability. This can be achieved by bioengineering and formulation of rhEs protein as a sustained release product. Point mutation of rhEs (P125A) leads to increasing of bioactivity compared with wild–type of hEs^91^ and some studies have established the efficiency of this bioengineering modification. Therefore, the more effective anti-angiogenesis inhibitors can be produced by molecular modification and structure/function analysis. Improving the bioactivity and localization of mutant endostatin were achieved via adding an integrin-targeting RGD moiety to N-terminal of mutant endostatin,^66,55^ because an additional sequence of NGR motif was generated with affinity to endothelial cell. The mutant and wild-type endostatin has short serum half-life that reduces effective concentration at the tumor and in turn necessitates frequent administration or continuous injection. This deficiency can be resolved by slow deliver formulation via fusing to anti-HER2 IgG or heavy-chain of IgG.^55,96^ Targeting antiangiogenic agents by antibody is a useful strategy that can be used for other tumor targets via replacement with other antibody specificities/variable domains.

Since the discovery of hEs, adding 9 amino acids to N-terminal of rhEs (endostar) is the best valuable molecular bioengineering of hEs, Because of increasing proteolytic resistance and thermos stability.^90^ The same as point mutated Es, endostar has short half- life in the plasma**.** There have been many attempts to increase the stability of endostar based on the use of new drug delivery carrier with well-controlled release form. Treatment of tumor using targeted therapy based on nanomaterial and nanotechnology is a very useful strategy in cancer therapy. The loaded endostar by PLGA and PEG-PLGA nanoparticles and gold nano-shell particles, showed better antitumor activity than conventional endostar because of sustained releasing and longer half –life in target tumor.^94,95,97^ Additionally, PEG-PLGA is a very useful carrier for encapsulation and distribution of endostar than other nanoparticle.^95^ PEG-PLGA nanoparticles can preserve sufficient concentrations of endostar in plasma and tumor; thereby improve its anti-cancer activity. Briefly, PEG-PLGA nanoparticles can be an effective carrier for protein drugs.

## Ethical Issues

Not applicable.

## Conflict of Interest

The authors declare no conflict of interest in this study.
